# Extract of Belladonna in Paraphimosis

**Published:** 1842-01

**Authors:** 


*Extract of Belladonna in Paraphimosis.*—A child, three years and a half old, had a severe paraphimosis; the glans red, swollen, and tender; the prepuce strongly drawn back, forming a thick and apparently adherent ring, the constriction of which completely stopped the circulation. This state had lasted eight days, and the sufferings were extreme. Reduction being impossible, leeches to the perineum and hypogastrium, cooling drinks, emollient enemata, cataplasms, lotions and hip baths were all tried, but with little good effect. All the symptoms were aggravated, the glans became bluish, and gangrene was threatened, when M. Mignot employed frictions round the glans with an unguent, composed of thirty parts of simple cerate and twelve of extract of belladonna. Under the influence of this remedy, the constriction relaxed, the tissues resumed their normal appearance; no loss of substance or suppuration followng.

The second patient had acute balanitis following gonorrhœa; the paraphimosis was severe. The patient would not submit to the operation; the belladonna was applied with complete success. It was given with the like effect in a case of paraphimosis, accompanied by chancres and sympathetic bubo. In three days the cure was complete.—*Brit. and For. Med. Rev.*

